# One-site polarity switch enhances catalytic efficiency and stability of d-allulose 3-epimerase via flexibility–rigidity rebalancing

**DOI:** 10.1038/s41598-026-54479-z

**Published:** 2026-05-30

**Authors:** Fina Amreta Laksmi, Yudhi Nugraha, Kenny Lischer, Khaerunnisa, David Herawan, Ario Betha Juanssilfero, Des Saputro Wibowo, Isa Nuryana, Syahputra Wibowo, Keni Vidilaseris, Mulyorini Rahayuningsih

**Affiliations:** 1 Directorate of Laboratory Management, Research Facilities, and Science Technology Parks, Deputy for Infrastructure Research and Innovation, National Research and Innovation Agency, Jalan Raya Bogor KM 46, Cibinong, Bogor, West Java 16911 Indonesia; 2 Research Center for Molecular Biology Eijkman, National Research and Innovation Agency, Jalan Raya Bogor KM 46, Cibinong , Bogor, West Java 16911 Indonesia; 3 Bioprocess Engineering, Department of Chemical Engineering, Faculty of Engineering, Universitas Indonesia, Depok, West Java 16424 Indonesia; 4 Research Center of Biomedical Engineering, Faculty of Engineering, Universitas Indonesia, Depok, West Java 16424 Indonesia; 5 Biotechnology Program, Graduate School, IPB University, Bogor, West Java Indonesia; 6 Chemical Engineering, Department of Chemical Engineering, Faculty of Engineering, Universitas Indonesia, Depok, West Java 16424 Indonesia; 7 Research Center for Environmental and Clean Technology, National Research and Innovation Agency, Jalan Raya Bogor KM 46, Cibinong, Bogor, West Java 16911 Indonesia; 8 Research Center for Applied Microbiology, National Research and Innovation Agency, Jalan Raya Bogor KM 46, Cibinong, Bogor, West Java 16911 Indonesia; 9 Molecular and Integrative Biosciences Research Programme, Faculty of Biological and Environmental Sciences, University of Helsinki, 00014 Helsinki, Finland

**Keywords:** d-allulose 3-epimerase, *Arthrobacter psychrolactophilus*, d-allulose, Rational mutation, Catalytic activity, Thermostability, Biochemistry, Biophysics, Chemistry, Structural biology

## Abstract

The structural foundation of enzyme efficiency frequently resides in the subtle equilibrium between localized flexibility and overarching rigidity. This study presents evidence that a singular amino acid alteration (E35H) in *Arthrobacter psychrolactophilus* d-allulose 3-epimerase (ApDAEase) enhances catalytic efficiency by 1.4 times relative to the wild-type, attributed to a 1.3-fold reduction in *K*_m_ and a 1.1-fold elevation in *k*_cat_. Structural insights were obtained from models predicted by AlphaFold in conjunction with molecular dynamics simulations. These findings indicated that the E35H substitution diminished the local hydrogen-bond network at residue 35, reducing interactions from six to five, which consequently loosened constraints within the active site. Analysis of RMSF revealed that localized flexibility was heightened at residues 35 and 6, whereas global fluctuations exhibited a decrease, aligning with the stabilization of the scaffold. Furthermore, a newly proposed interaction between E152 and substrate O1 offered an extra anchoring effect. CD spectroscopy and thermal unfolding analyses revealed a slight enhancement in thermostability, with a melting temperature of 94.3 °C compared to 92.6 °C observed in the wild-type. Collectively, these findings highlight residue 35 as a critical functional hotspot, where the modulation of polarity effectively reestablishes a balance between flexibility and rigidity, thereby providing valuable predictive insights into the mechanism of improved catalysis in DAEase.

**Supplementary Information:**

The online version contains supplementary material available at 10.1038/s41598-026-54479-z.

## Introduction

In recent decades, there has been a dramatic global increase in the prevalence of metabolic disorders such as obesity, type 2 diabetes mellitus, and cardiovascular disease. This surge is strongly associated with modern dietary patterns characterized by an excessive intake of foods high in sugar and fat. The widespread consumption of such energy-dense, nutrient-poor foods has resulted in significant public health challenges and escalating healthcare costs worldwide^1,2^.

This growing health crisis has stimulated considerable interest in the development of low-calorie sweeteners that can replace traditional sugars without sacrificing consumer-desired sensory properties. Among these, d-allulose (also known as d-psicose), a C-3 epimer of d-fructose, has gained substantial attention. d-allulose not only possesses an appealing taste profile but also offers proven health benefits, including anti-obesity, anti-hyperglycemic, and anti-inflammatory effects, making it an attractive alternative for both the food industry and health-conscious consumers^3^.

The enzymatic production of d-allulose through the action of d-allulose 3-epimerase (DAEase) is generally preferred over chemical synthesis. Enzymatic approaches offer higher substrate specificity, better environmental sustainability, and milder operational conditions^4,5^. DAEase catalyzes the reversible epimerization of d-fructose into d-allulose by changing the stereochemistry at the C3 position of the fructose molecule. However, the majority of naturally occurring DAEases exhibit limited thermostability and suboptimal catalytic efficiency, rendering them less suitable for cost-effective and large-scale industrial applications^6,7^.

Continuous efforts have thus been made to identify and engineer DAEase variants with improved industrial traits. *Arthrobacter psychrolactophilus*-derived DAEase (ApDAEase) has recently emerged as a promising candidate due to its notable thermostability. When heterologously expressed in *Escherichia coli* BL21 Star (DE3), ApDAEase demonstrated optimal activity at elevated temperatures (70 °C, pH 8.5, supplemented with 1 mM Mg²⁺), with a 27% conversion rate from 500 mg/L d-fructose^8^. Comparative sequence analyses have revealed a high degree of identity (88.24%) between ApDAEase and DAEase from *Arthrobacter globiformis*, along with some distinct structural characteristics^9^. Despite this inherent thermostability, further improvements in catalytic efficiency are still needed to render ApDAEase more commercially viable.

To address these challenges, various protein engineering strategies, including structure-based site-directed mutagenesis, saturation mutagenesis, and semi-rational engineering, have been utilized to optimize DAEase enzymes from different sources. For example, structure-guided mutagenesis of the enzyme from *Thermoclostridium caenicola* resulted in significant gains in thermostability and optimal temperature range, while other variants from *Christensenellaceae bacterium* and *Thermogutta terrifontis* exhibited substantial improvements in catalytic efficiency and pH tolerance^6,7,10^. In light of recent advancements, it is noteworthy that the majority of engineering initiatives have predominantly concentrated on enhancing thermostability or catalytic efficiency via sequence-based or structural methodologies. However, there has been a relative lack of focus on the modulation of the electrostatic and polarity characteristics within the active-site microenvironment. The modulation of these properties is of significant importance, as electrostatic interactions and residue polarity play a crucial role in substrate recognition, binding orientation, and transition state stabilization, while facilitating the fine-tuning of local flexibility while maintaining the overall structural integrity^11,12^. Nevertheless, engineering approaches targeting the modulation of charge and polarity of residues surrounding the active site in ApDAEase have not yet been reported.

This study seeks to address this gap by employing a site-directed mutagenesis strategy. By selectively modifying the charge and polarity of amino acid residues proximal to the active site, we aim to enhance substrate binding and transition state stabilization, thereby improving catalytic activity while maintaining enzyme stability. The resulting ApDAEase mutants were expressed in *E. coli*, purified, and rigorously characterized for their biochemical properties. Unlike previous efforts that primarily emphasized on sequence conservation or predicted structural features, our approach integrates these approaches with the mutational selection based on comprehensive electrostatic and polarity mapping. This work thus represents a novel methodological framework for the rational design and engineering of DAEase biocatalysts, contributing to the development of more efficient and competitive processes for industrial d-allulose production.

## Materials and methods

### Bacterial strains, plasmid, and medium

*Escherichia coli* BL21 Star (DE3) served as the host for the site-directed mutagenesis and protein expression of ApDAEase. The organism was cultivated in Luria Broth medium (LB), with the addition of 30 µg/mL kanamycin. The plasmid pET-28a(+) (Novagen, Germany), which harbors both the wild-type and mutant strains of *Arthrobacter psychrolactophilus*, served as the expression vector in this study. A pre-culture of the transformant carrying an expression vector was conducted using LB–kanamycin supplemented with 0.4% glucose. Reagents of analytical grade were employed to conduct the enzymatic reactions. All reagents were sourced from esteemed suppliers, including Merck, TCI, Himedia, among others.

### Selection of potential amino acid residues in the active site area

The subsequent methodology for identifying candidate amino acid residues within the binding pocket was executed utilizing AlphaFold 3, adhering to advanced structural prediction techniques^13^. To evaluate the evolutionary conservation of these residues, a multiple sequence alignment (MSA) of ApDAEase and its homologs was performed using Clustal Omega (EMBL-EBI; https://www.ebi.ac.uk/jdispatcher/msa/clustalo), and the results were visualized using the ESPript 3.2 server. The model structure underwent visualization and analysis through the utilization of PyMol software version 3.1.6.1^14^. The crystal structure of *Arthrobacter globiformis* d-allulose 3-epimerases (AgDAEase) served as the template (PDB ID: 5ZFS)^15^, to examine the amino acid residues located in the active site region of ApDAEase. The superimposition of the target protein (ApDAEase) onto its template (AgDAEase) was conducted utilizing PyMol. The selection of the AgDAEase crystal structure was informed by the significant identity of 88.24% observed between AgDAEase and ApDAEase. All residues adjacent to O3, O4, O5, and O6 of d-fructose were chosen for additional examination. Furthermore, the thermodynamic impact of site-specific mutations was predicted using the MaestroWeb server (https://pbwww.services.came.sbg.ac.at/maestro/web) to calculate changes in Gibbs free energy (ΔΔG), providing an initial screening for structural stability.

### Site directed mutagenesis

Site directed mutagenesis was conducted following the protocol of the PrimeSTAR^®^ GXL DNA Polymerase Kit (Takara, Japan) using the whole plasmid PCR method^16^, with several modifications. The synthesis of the mutant strands was achieved through PCR, utilizing 125 ng of two complementary mutagenic primers (Table S1) alongside 10 ng of recombinant pET-28a(+)–ApDAEase as the template. The template consisted of the pET-28a(+) vector (~ 5.3 kbp) and the 870 bp ApDAEase gene insert, totaling approximately 6.2 kbp. The designed primers aimed to induce mutations by substituting the original amino acid residue at the specified mutation site (H6D, E35H, S63D, V105D, M110H, and M110D). The PCR cycle implemented for the amplification of the entire 6.2 kbp recombinant plasmid consisted of an initial denaturation at 98 °C for 1 min, succeeded by 18 cycles of denaturation at 98 °C for 1 min, annealing at 68 °C for 15 s, and an extension at 68 °C for 7 min (optimized for the ~ 6.2 kbp length), concluding with a final extension at 68 °C for 7 min. The amplified mutant strand underwent purification utilizing the NucleoSpin™ Gel and PCR Clean-up Kit (Takara, Japan) and was subsequently treated with DpnI (NEB, England) at 37 °C for 1 h to effectively digest the methylated parental template. The PCR product, treated with DpnI, was introduced into *E. coli* BL21 Star (DE3) and incubated overnight at 37 °C on an LB–kanamycin agar plate.

### Expression and purification of ApDAEase

Recombinant *E. coli* BL21 Star (DE3) containing pET-28a(+)–ApDAEase wild-type and pET-28a(+)–ApDAEase mutant was pre-cultured in LB–kanamycin medium supplemented with 0.4% glucose (w/v). A 1% aliquot (v/v) of the pre-culture was subsequently introduced into 400 mL of LB-kanamycin medium and incubated at 37 °C with shaking at 165 rpm. The ApDAEase was overexpressed overnight at 37 °C with shaking at 165 rpm, following the addition of 0.2 mM IPTG when the OD600 reached 0.8–1.0. The process of centrifugation was conducted at 8000 rpm for a duration of 8 min at a temperature of 4 °C to collect the cells. The pellet was resuspended in a 50 mM Tris-HCl buffer at pH 8.0 and subjected to sonication six times, with intervals of 30 s on and 30 s off while kept on ice. The supernatant, which contains the enzyme (crude extract), was isolated from the cell debris through centrifugation at 14,000 rpm for 15 min at a temperature of 4 °C.

To achieve the purification of ApDAEase, a HisTrap HP column (Cytiva, USA) was utilized in conjunction with the AKTA Prime Plus Liquid Chromatography System (Cytiva, USA). The column was equilibrated using buffer A, which consisted of 20 mM sodium phosphate, 500 mM NaCl, and a pH of 7.4, supplemented with 20 mM imidazole. Subsequently, the crude extract was automatically introduced into the column for processing. A solution of Buffer B, comprising 20 mM sodium phosphate, 500 mM NaCl, and adjusted to pH 7.4, was introduced into the column using a linear gradient of imidazole ranging from 20 to 500 mM, at a flow rate of 1 mL min^− 1^. The imidazole was eliminated through dialysis of the combined fractions using a solution composed of 50 mM Tris-HCl buffer (pH 8.0), 100 mM NaCl, and 10% glycerol.

The method of Laemmli (1970)[17] was employed to conduct sodium dodecyl sulfate-polyacrylamide gel electrophoresis (SDS-PAGE) on a 12% gel, aimed at analyzing the molecular mass of purified wild-type and mutant ApDAEase. Western blotting was conducted following the manual protocol of the HisProbe^TM^-HRP Conjugate kit (ThermoFisher, USA) and utilizing the Tetra Blotting Module (BioRad, USA), with the chromogenic substance 3,3’,5,5’-tetramethylbenzidine (TMB) employed for band visualization. The determination of protein concentration was conducted utilizing the bicinchoninic acid (BCA) method, employing bovine serum albumin as the standard for protein comparison.

### Enzyme activity

The enzyme activities of the wild-type and mutants ApDAEase were assessed according to the methodology established by Laksmi et al. (2022)[8], incorporating several modifications to quantify the production of d-allulose from d-fructose. The typical procedure involved a total mixture volume of 1 mL, comprising 50 mM Tris-HCl buffer (pH 8.5), 100 mM substrate, 1 mM MgCl_2_, and 100 µg of enzyme. This mixture was incubated at 70 °C for 30 min, after which the reaction was terminated by boiling for 5 min. The enzyme unit is characterized by the total count of enzymes facilitating the production of 1 µmol d-allulose per minute under optimal conditions of pH and temperature, specifically at 70 °C and pH 8.5. The analysis of d-allulose is conducted using both quantitative and qualitative methods through HPLC (Shimadzu, Japan), which is outfitted with a refractive index detector and an HILICpak VG-50 4E Column (Shodex). The temperature was maintained at 40 °C, while 80% (v/v) acetonitrile was delivered at a flow rate of 1 mL per minute. The application of HPLC enables precise separation and quantification of the substrate (d-fructose) and the product (d-allulose) within a complex enzymatic reaction mixture, utilizing their distinct retention times for accurate measurement.

### Kinetic parameters

The kinetic parameters were assessed using d-fructose as the substrate, within a concentration range of 5 to 1920 mM. The reaction mixture consisted of 50 mM Tris-HCl buffer (pH 8.5), substrate, and 1 mM MgCl_2_, which was incubated with 100 µg of enzyme at 70 °C for a duration of 30 min. The values of *K*_m_ (mM) and *k*_cat_ (min^− 1^) were determined utilizing the Michaelis–Menten equation through the application of GraphPad Prism 10 (GraphPad software).

### Circular dichroism

To assess the thermal stability of both wild-type and mutant ApDAEase, a Jasco J-1500 CD spectrometer equipped with a Peltier cell holder and a PTC-510 temperature controller was employed to evaluate the melting of ApDAEase at a concentration of 0.2 mg ml^− 1^ in a 50 mM Tris-HCl buffer at pH 8.5. The data were gathered at high-tension voltage levels below 600 V. DAEase underwent dialysis using a 50 mM Tris-HCl buffer (pH 8.5) supplemented with 2% glycerol for an overnight period. The protein concentration following dialysis was quantified utilizing the bicinchoninic acid (BCA) assay, employing bovine serum albumin as the standard for protein measurement. Thermal melting was conducted within a temperature range of 30 to 90 °C, employing a scan rate of 30 °C per hour, utilizing a cuvette with a pathlength of 0.1 cm. The ellipticity at 216 nm served as a means to monitor alterations in the secondary structure.

### Molecular docking and molecular dynamics (MD) simulations

The three-dimensional structure of ApDAEase was generated utilizing Alpha Fold 3^13^, which offers high-confidence predictions for both backbone and side-chain configurations. Given the absence of an experimental structure for ApDAEase, the AlphaFold model was utilized as a framework for conducting molecular dynamics simulations and analyzing hydrogen bonds. Although predictions made by AlphaFold cannot replace crystallographic or cryo-EM data, they offer a dependable foundation for comparative mutational analyses, especially when corroborated by experimental biochemical and biophysical validation^18^.

The structural modeling of ApDAEase was performed using Alpha Fold 3 (https://alphafoldserver.com), configured as a tetramer, and subjected to structural prediction simulations. The amino acid sequence was retrieved from the NCBI database (Accession No. WP_110484351.1), which corresponds to the nucleotide sequence under accession NZ_QJVC01000003.1. The protein record was also cross-referenced with the UniProtKB database under the entry code A0A2V5JHE2. Each prediction was carried out without templates, employing automatic alignments and the default number of recycles to generate five relaxed structural models. AlphaFold will provide a per-residue predicted local difference test (pLDDT) score as a confidence metric and a predicted template modelling (pTM) score for each model^19^. The resulting structure is then exported in PDB format for further analysis.

Molecular docking was performed using DiffDock-Web, accessed through HuggingFace (running on an NVIDIA Tesla T4 GPU backend)^20^. The PDB file of the predicted structure of the ApDAEase protein was uploaded along with the SDF file of the d-fructose substrate ligand obtained from PubChem (PubChem ID: 2723872). The docking results were then visualised using the PyMOL Version 3.1.6.1^14^, to analyse the interactions between key residues and the d-fructose substrate.

Molecular dynamics (MD) simulations were performed using the GROMACS v2023.1 package, following a modified protocol based on Paul et al. (2025)[21]. Initial three-dimensional structures of the wild-type ApDAEase and the E35H mutant were generated using Alpha Fold 3. The protein components were parameterized with the AMBER ff19SB force field, while the ligand was modeled using the General Amber Force Field 2 (GAFF2). Ligand partial charges were assigned using the AM1-BCC method via Antechamber. Topology and coordinate files were prepared using AmberTools22 and subsequently converted to GROMACS-compatible format through the ParmEd library to ensure parameter consistency. Each system was solvated in a cubic box with TIP3P water molecules, maintaining a minimum distance of 1.2 nm between the protein and the box edges. The systems were neutralized by adding sodium and chloride ions to reach a physiological concentration of 0.15 M NaCl. Energy minimization was conducted using the steepest descent algorithm until the maximum force was less than 1000 kJ mol^−1^nm^− 1^. System equilibration was carried out in two successive stages: a NVT ensemble followed by NPT ensemble. The temperature was maintained at 300 K using the V-rescale thermostat, and the pressure was regulated at 1.0 bar using the Parrinello-Rahman barostat. Finally, a 50 ns production MD run was performed with a 2 fs integration time step. Long-range electrostatic interactions were treated using the Particle Mesh Ewald (PME) method, and van der Waals interactions were calculated with a 1.0 nm cut-off. All bonds involving hydrogen atoms were constrained using the LINCS algorithm, and simulations were performed under 3D periodic boundary conditions (xyz). The analysis of the MD trajectories was conducted utilizing GROMACS built-in modules to evaluate structural stability and interaction dynamics. Structural stability was assessed through Root-Mean-Square Deviation (RMSD) analysis, while local flexibility was determined by Root-Mean-Square Fluctuation (RMSF) calculations. The number of hydrogen bonds, as well as specific interatomic distances and interaction angles, were calculated throughout the trajectory using the gmx hbond, gmx distance, and gmx angle modules. Furthermore, hydrogen bond occupancy was analysed using the Hydrogen Bonds plugin in VMD version 1.9.4 (2b1), employing a geometric distance cutoff of 0.35 nm and an angle cutoff of 30°. To facilitate comparative structural analysis, RMSF values were converted into pseudo-B-factors using the equation RMSF^2^ = B × 3/8/ π^2^^22^ and color-coded onto the protein backbone. Additionally, the physicochemical environment of the targeted sites was characterized by mapping surface electrostatic potentials using the Adaptive Poisson-Boltzmann Solver (APBS) plugin and surface hydrophobicity via a normalized consensus scale within PyMOL.

## Results

### Structure modeling of ApDAEase

**Fig. 1:**
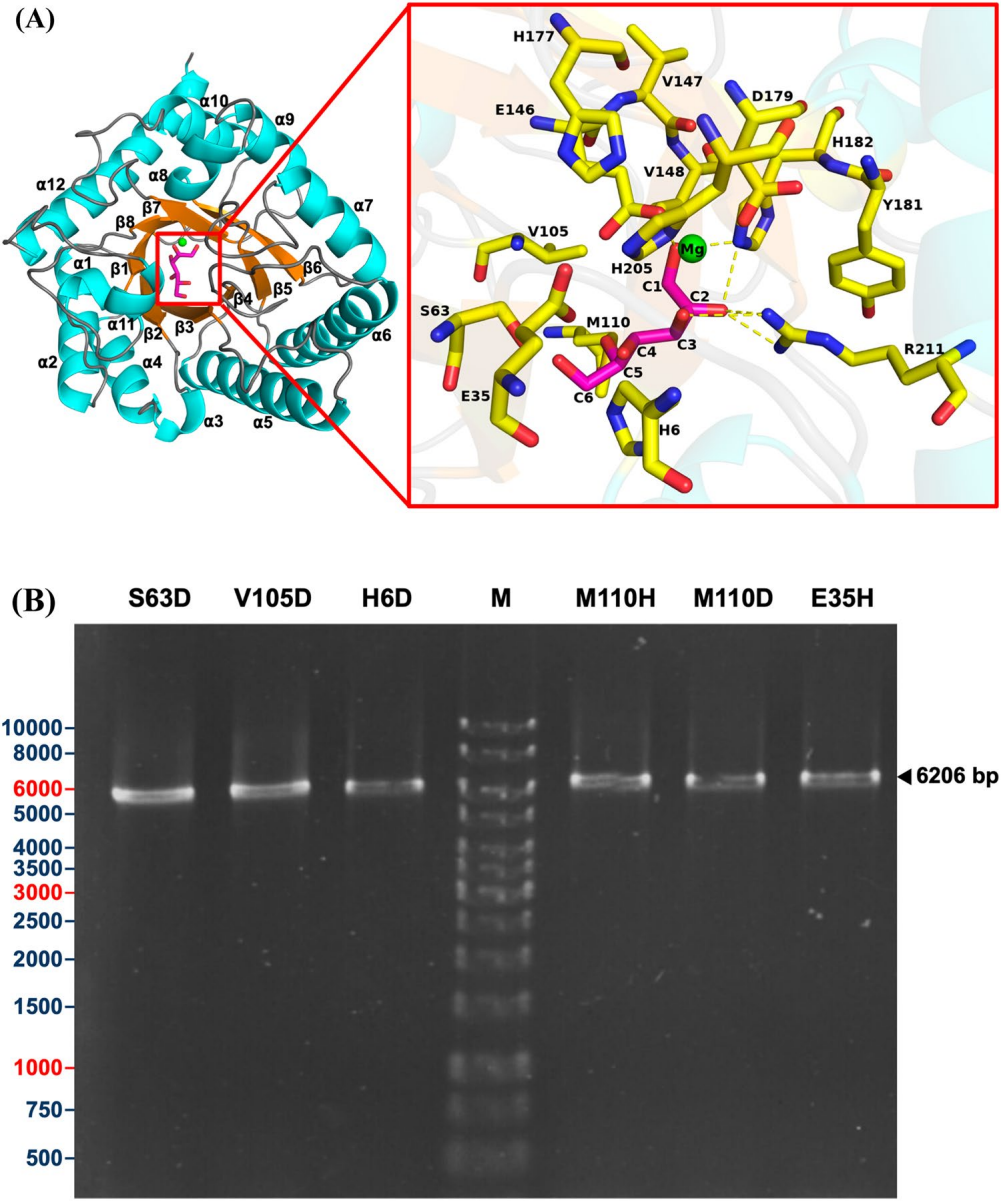
Structural model and verification of ApDAEase mutant candidates. (**A**) Ribbon cartoon structure model of ApDAEase with its key residues. The alpha-helix structure is shown in blue, the beta-sheet in orange, and the loop in gray. The yellow sticks represent the key residues, while the magenta sticks represent the substrate d-fructose. The hydrogen bond interactions between the key residues and the O1, O2, and O3 atoms of d-fructose are depicted as yellow dashed lines. (**B**) Electrophoresis of PCR products showed that the mutant candidate was successfully amplified. Lane M: DNA ladder (GeneRuler 1 kb DNA ladder). The triangle symbol indicates the targeted plasmid size.

The latest advances in structure of protein prediction, particularly through AlphaFold, have notably improved the accuracy of enzyme modeling directly from primary amino acid sequences. This study presents the de novo prediction of the three-dimensional structure of ApDAEase utilizing AlphaFold, independent of any homologous structural templates. The high-confidence model demonstrated that ApDAEase consists of eight β-strands, twelve α-helices, and various loop regions (Fig. 1A). At its conserved core, the monomer exhibits the classic TIM-barrel (β/α) 8 structure, which is a defining feature of this enzyme family. The anticipated tetrameric assembly of ApDAEase showed a pTM (predicted TM-score) value of 0.96 and an ipTM (interface predicted TM-score) value of 0.96, reflecting a high level of confidence and precision in the prediction of its quaternary structure. Molecular docking was conducted using Diffdock software to derive the model of the enzyme-substrate complex.

### Construction of rational ApDAEase variants

The residues E146, D179, H205, and E240 (Fig. 1A), essential for the coordination of the Mg²⁺ cofactor, exhibited strict conservation across all documented DAEases. The hydroxyl group located at the O1 position of the substrate forms two hydrogen bonds with E146 and one hydrogen bond with H182. At the O2 position, a hydrogen bond is formed with H182 via a single interaction, while R211 engages in two distinct interactions with the O2 atom. The O3 atom participates in an additional hydrogen bond with R211, as well as two hydrogen bonds with E240. Conversely, there are no residues that engage with the O5 and O6 atoms. In light of these observations, several residues facilitating interactions with the substrate in proximity to this area demonstrated a significant level of conservation. The retention of these residues indicates their essential role in catalytic function or the upkeep of the protein’s structural stability, making them inappropriate targets for modification. The findings are supported by a comprehensive multiple sequence alignment (MSA) analysis (Fig. S1), indicating that the residues involved in metal coordination (E146, D179, H205, and E240), along with the active-site residues that directly interact with the substrate (H182 and R211), exhibit a high degree of conservation among DAEase homologs. This conservation underscores their critical functions and provides a rationale for their exclusion from mutagenesis. Consequently, for the purpose of modification aimed at enhancing activity while ensuring stability, residues were chosen that are situated near the O4, O5, and O6 atoms. These residues do not engage in direct interactions with the substrate and remain within the vicinity of the binding pocket. The residues identified according to these parameters include H6, E35, S63, V105, and M110 (Fig. 1A). Subsequently, the chosen residues are replaced with those exhibiting contrasting the electrostatic charge or transitioned from non-polar to polar states. In accordance with this approach, the initial residues underwent mutations to H6D, E35H, S63D, V105D, M110D, and M110H.

In the subsequent phase, site-directed mutagenesis of the specified residues was conducted to enhance the catalytic activity. The mutant variants were generated via whole plasmid PCR utilizing the recombinant pET-28a(+)–ApDAEase plasmid as the template. The amplification of the ApDAEase gene was achieved, resulting in a singular, clearly defined band as illustrated in (Fig. 1B). The detected band represents the ~ 6.2 kbp construct, comprising both the plasmid backbone and the ApDAEase insert gene.

### Expression and purification of mutant variants

**Fig. 2:**
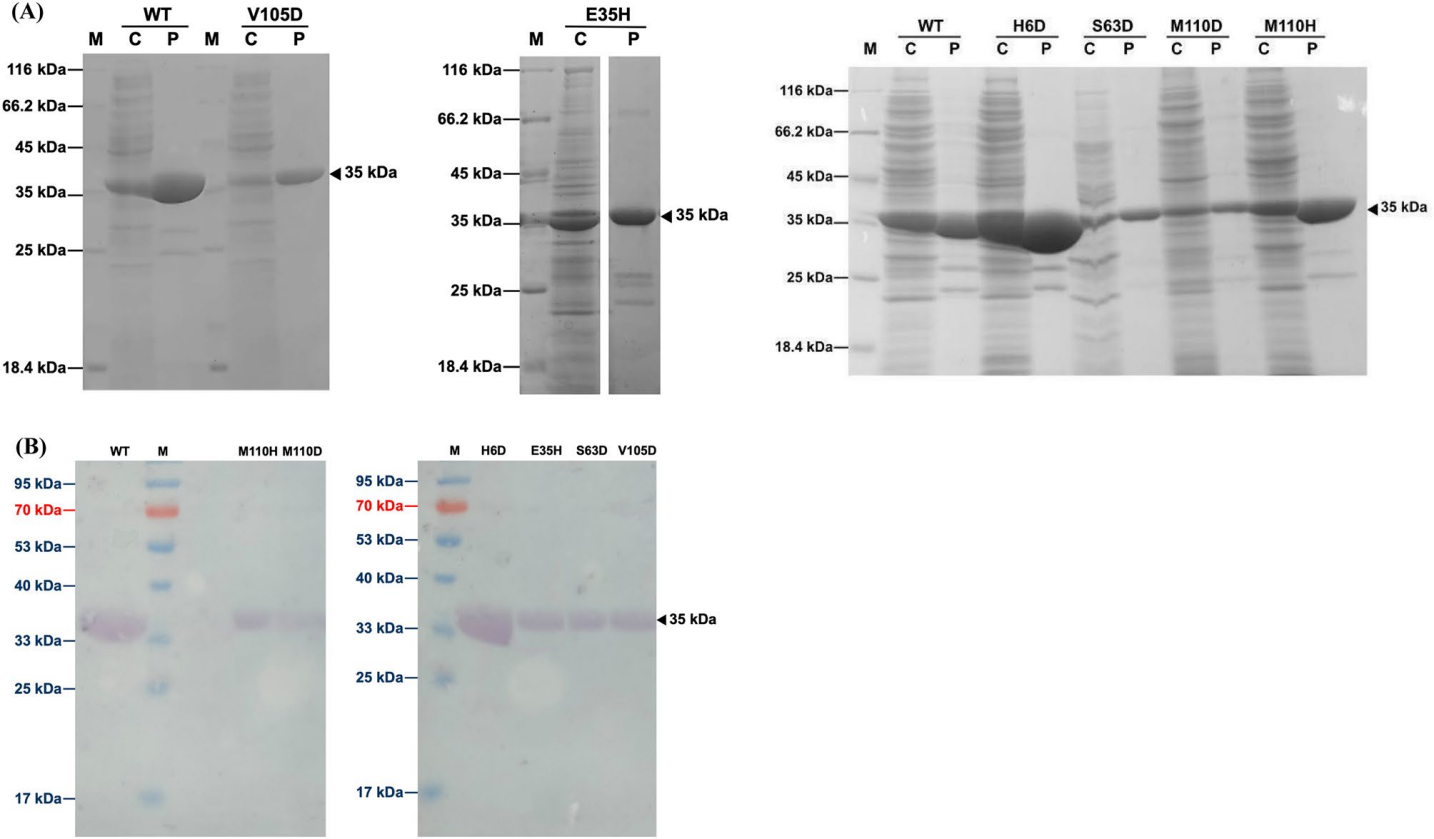
SDS-PAGE and western blot of ApDAEase mutants. (**A**) SDS-PAGE analysis results of ApDAEase mutants purification. Lane M: protein ladder, lane C: crude extract, lane P: purified protein. For presentation purposes, lanes in the right panel were rearranged from their original positions on the same gel and are delineated by white space. (**B**) Western blot analysis of ApDAEase mutants. The triangle symbol indicates the target protein band. Full-length gels and blots are provided in the Supplementary File.

To investigate the biochemical characteristics of ApDAEase wild-type and mutant variants, purification of the soluble fraction was conducted through affinity chromatography utilizing a HisTrap column (1 mL). The purity of both wild-type and mutant variants of ApDAEase is illustrated in (Fig. 2A). The high purity of ApDAEase wild-type and mutant variants was successfully achieved, with the molecular weight of each subunit projected to be approximately 34 kDa, consistent with the molecular weight derived from the amino acid sequence. Subsequent analysis revealed that western blotting utilizing HisProbe™-HRP Conjugate corroborated the expression of ApDAEase, yielding a comparable result at approximately 34 kDa (Fig. 2B).

### Effect of modification on their biochemical properties

**Fig. 3:**
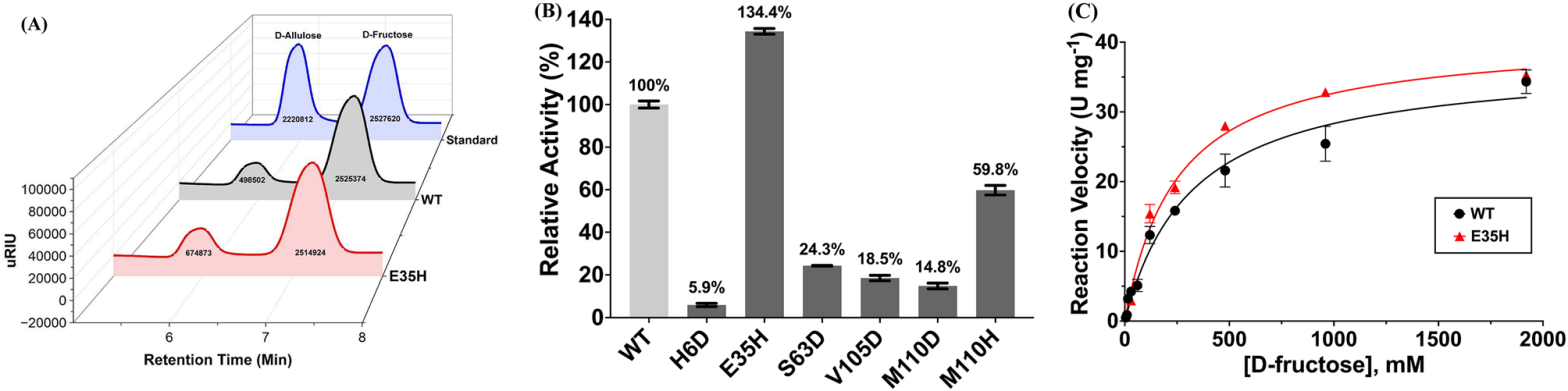
Enhanced catalytic activity of ApDAEase mutants toward d-fructose. (**A**) HPLC chromatograms comparing d-allulose production by ApDAEase wild-type (WT) and E35H mutant with standard retention times for d-fructose and d-allulose. (**B**) Relative catalytic activity of ApDAEase wild-type and various mutants toward d-fructose. (**C**) Michaelis-Menten plots of ApDAEase wild-type and E35H mutant toward d-fructose as substrate.

The quantitative analysis of the catalytic activity of ApDAEase in facilitating the conversion of d-fructose to d-allulose was conducted using HPLC. The retention times recorded for d-allulose and d-fructose were 5.9 and 7.2 min, respectively (Fig. 3A). The variants H6D, S63D, V105D, M110D, and M110H exhibited a significant decline in catalytic activity (Fig. 3B). Notably, the H6D variant demonstrated a nearly complete loss of catalytic function when compared to the wild-type, with a 94.1% decrease in activity post-modification. The other variants followed with reductions of up to 75.7%, 81.5%, 85.2%, and 40.2%, respectively. Only variant E35H exhibited an enhancement in activity of approximately 34.4% following modification.

Subsequently, an investigation into the kinetic properties of the E35H variant was conducted to elucidate the underlying reasons for its enhanced catalytic activity. The investigation into the impact of mutations on kinetic properties was conducted using the Michaelis–Menten plot, as illustrated in Table 1 and (Fig. 3C). A Michaelis–Menten kinetic plot that contrasts the enzymatic activity between the wild-type (WT) enzyme and the E35H variant is shown in (Fig. 3C). The E35H variant exhibits a more pronounced initial increase and achieves greater reaction velocities at smaller substrate concentrations in comparison to the wild-type, as demonstrated by the upward shift of the red curve in relation to the black curve. The findings indicate that the use of d-fructose as the substrate led to a 1.3-fold decrease in the *K*_m_ values of E35H, implying an increased affinity for the substrate. Simultaneously, the *k*_cat_ values demonstrated a 1.1-fold increase, resulting in a 1.4-fold improvement in catalytic efficiency (*K*_m_/*k*_cat_) compared to the wild-type.

**Table 1:** Kinetic parameters of wild-type (WT) and E35H ApDAEase.

ApDAEase	Specific Activity (U/mg)	K_m_ (mM)	k_cat_ (min⁻¹)	k_cat_/K_m_ (min⁻¹·mM⁻¹)
Wild-type	37.44 ± 1.52	321.3 ± 37.71	1252 ± 50.94	3.90 ± 0.48
E35H	40.82 ± 1.00	248.8 ± 18.78	1365 ± 33.42	5.49 ± 0.44

Kinetic values were determined using [d-fructose] under optimal conditions. The data highlights the comparison of catalytic capacity (*k*_cat_) and catalytic efficiency (*k*_cat_/*K*_m_ ) between the two variants. Data are presented as the mean ± standard error (SE) derived from triplicate independent experiments (*n* = 3).

### Structural comparison of wild-type and E35H and the effect on their thermal stability

**Fig. 4:**
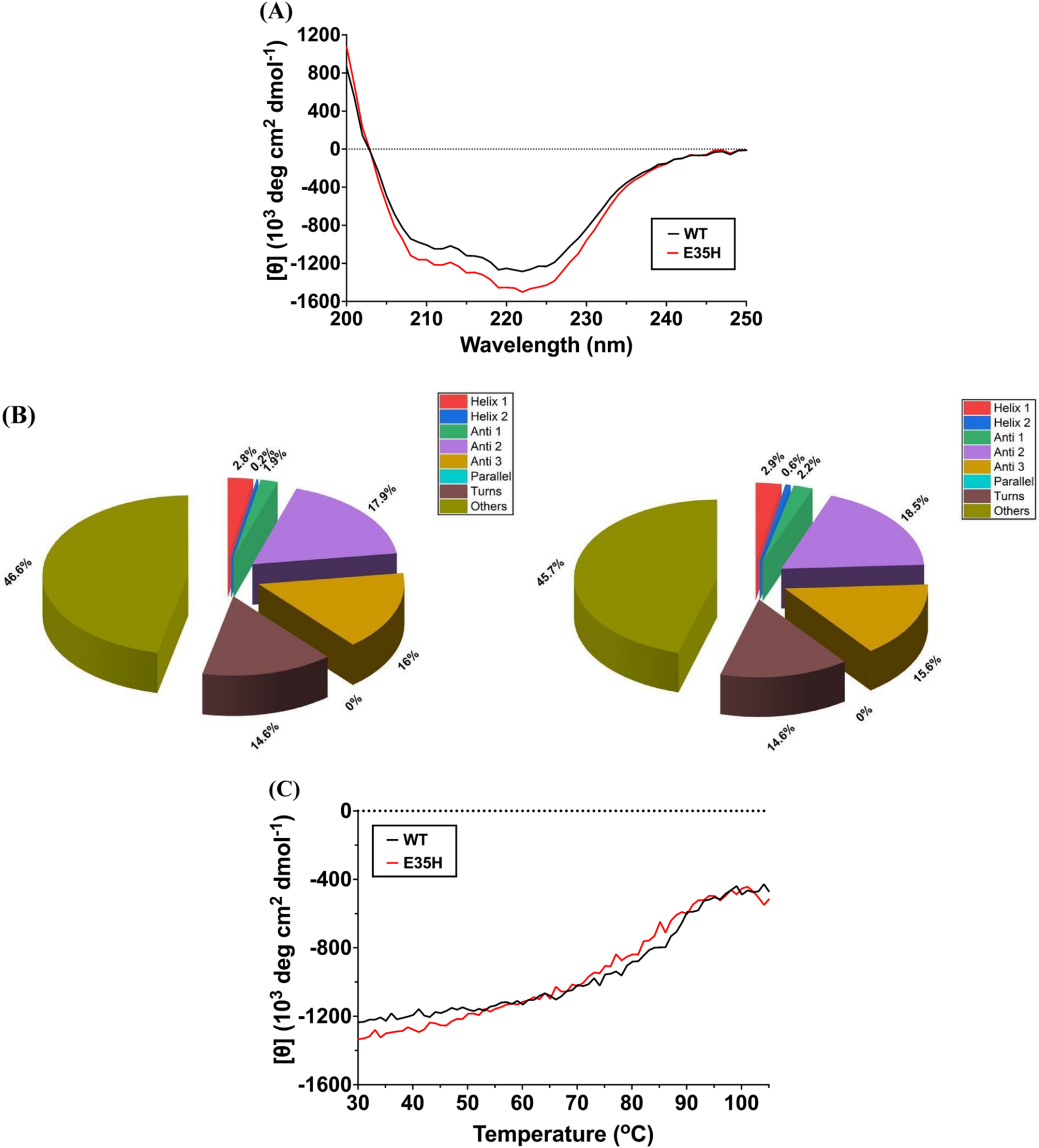
Circular dichroism (CD) spectra analysis of ApDAEase wild-type (WT) and mutant E35H. **(A)** Far-UV CD spectra of ApDAEase wild-type (black) and E35H mutant (red) in the wavelength range of 200–250 nm, showing the overall secondary structure composition. **(B)** Quantitative secondary structure content of ApDAEase wild-type (left) and E35H mutant (right) predicted from CD spectra. **(C)** Thermal denaturation profiles of ApDAEase wild-type (black) and E35H mutant (red) monitored by CD over the temperature range of 30–100 °C.

In order to assess the potential structural alterations induced by the mutation in the E35H variant, a comparative analysis of the conformation of the wild-type ApDAEase protein and the E35H variant was conducted through the examination of CD spectra (Fig. 4). The analysis of CD spectra reveals distinct profile variations between the wild-type and E35H variants, illustrated by black and red lines, respectively (Fig. 4A). The wild-type and E35H variants exhibit distinct negative ellipticity across the wavelength range commonly associated with protein secondary structure analysis, specifically between 200 and 250 nm. The E35H variant consistently exhibits a more pronounced negative ellipticity compared to the wild-type across these wavelengths, indicating an enhanced level of ordered secondary structure. The composition of secondary structures in both the wild-type (WT) and E35H variant, expressed as a percentage of the total content assessed through Beta Structure Selection (BeStSel™) software^23^, is shown in (Fig. 4B). The helix content demonstrates a significant rise from 3% in the wild-type to 3.5% in the E35H variant, suggesting that the mutation marginally improves helical propensity. The content of the antiparallel β-sheet exhibits a notable degree of stability following modification, rise from 35.8% in the wild-type to 36.3% in the E35H variant, indicating that this mutation does not substantially compromise the integrity of the β-sheet structure. The aggregate percentage of turns and additional structural elements (Turns + Others) diminishes from 61.2% in the wild-type to 60.3% in the E35H variant, indicating a nuanced transition of structural components from less organized configurations to more helix-dominant conformations in the mutant samples.

The thermal denaturation of the secondary structures in both the wild-type and E35H variant was evaluated by observing the reduction in ellipticity of the CD signal at 222 nm. The temperature-dependent CD signals in the far-UV region displayed characteristic sigmoidal curves, which are indicative of unfolding transitions, as shown in (Fig. 4C). The wild-type ApDAEase exhibited denaturation starting at approximately 60 °C, reaching complete unfolding at around 95 °C. In comparison, the E35H mutant exhibited a marginally earlier initiation of unfolding at around 55 °C, ultimately achieving total denaturation later, close to 100 °C. The Tm value of E35H, as determined by spectra manager™ (JASCO, Japan), is approximately 94.3 °C, indicating a slight increase in stability compared to the wild-type, which is around 92.6 °C. The supporting file, Table S2, shows thermal melting measurement data obtained from the Spectra Manager™ software (Jasco J-1500).

## Discussion

The optimization of d-allulose 3-epimerase (DAEase) for industrial use presents significant challenges, primarily because it requires the concurrent improvement of catalytic efficiency while ensuring thermal stability is preserved. DAEase from *Arthrobacter psychrolactophilus* demonstrates remarkable thermal stability, with a documented half-life at 55 °C of around 23.1 h Laksmi et al. (2022)[8], an essential factor for maintaining activity during extended industrial reaction conditions. Enzymes exhibiting enhanced thermostability are of significant interest in bioprocessing due to their ability to endure extreme conditions, including high temperatures. This resilience facilitates increased reaction rates and reduces the risks associated with microbial contamination, ultimately leading to enhanced process efficiency and improved product quality. Nonetheless, enhancing thermal stability frequently results in a more inflexible protein conformation, potentially limiting the dynamic adaptability required for effective substrate interaction, catalytic activity, and product liberation. The observed rigidity frequently results in a reduction of catalytic turnover and a decrease in overall enzymatic activity^24–26^. The flexibility of molecules remains essential, as it significantly influences enzyme catalysis. This is evident in the frequent reorganization of the active site during substrate binding, the formation of the transition state, and the release of products^11,27^. Effective protein engineering seeks to refine this equilibrium by improving targeted molecular interactions, especially in proximity to the active site and regions accessible to substrates, while avoiding a global rigidification of the protein structure^28^.

Recent advancements in computational protein modelling, especially with AlphaFold, have allowed for precise predictions of three-dimensional structures, thereby enabling a thorough evaluation of active site and microenvironment characteristics. AlphaFold produces comprehensive metrics, including the predicted TM-score (pTM) and the interface predicted TM-score (ipTM), which assess the reliability of the predicted overall structure and the interfaces between subunits, respectively^29^. The pTM-scores are measured on a scale ranging from 0 to 1, where values greater than 0.5 generally indicate that the predicted fold may resemble the structure^30^. In contrast, ipTM scores that surpass 0.8 are considered to be of high reliability, indicating exceptional predictive quality, while those falling below 0.6 are indicative of a likely unsuccessful prediction^13^. Our investigation revealed that the ApDAEase structure prediction achieved pTM and ipTM scores of 0.96, indicating a remarkably high level of confidence in the precision of the predicted monomeric fold and the assembly of the quaternary structure. The metrics discussed here hold significant importance in forecasting multimeric protein assemblies, enhancing the precision of predictions at protein interfaces that frequently play a crucial role in enzymatic stability and activity^31^.

Besides pTM and ipTM, pLDDT (predicted local distance difference test) is another important confidence value generated by AlphaFold. pLDDT is a measure of local confidence per residue. The pLDDT value has a scale ranging from 0 to 100, where a higher score indicates a higher level of confidence and generally indicates a more accurate structure prediction. pLDDT assesses the level of confidence by estimating the extent to which the predicted results will agree with the experimental structure. This value is obtained based on the local distance difference test C⍺ (IDDT-C⍺). This assessment method does not rely on structure superposition, but rather evaluates the accuracy of local interatomic distances^32^. pLDDT values above 90 are categorised as the highest level of accuracy, where both the mainframe (backbone) and side chains are generally predicted with high accuracy^33^. In the case of our study, almost all residues of ApDAEase wild-type and E35H mutant have scores above 90, as shown in the supporting file (Fig. S2), which indicates a very high level of confidence in both the backbone and side chains. Thus, the process of molecular docking and structure prediction becomes more reliable.

**Fig. 5:**
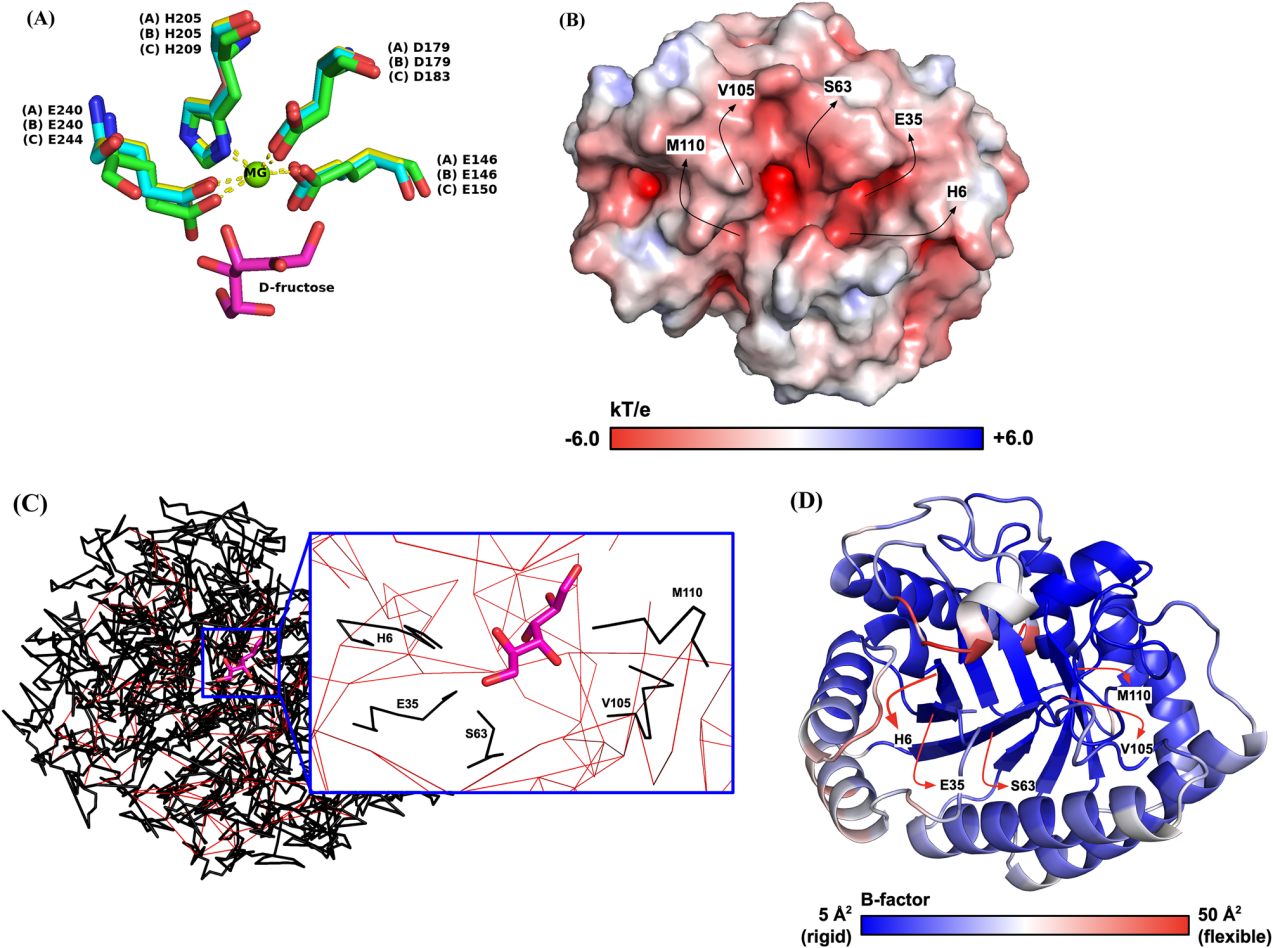
Comparative structural analysis of DAEase enzymes and physicochemical environment of the mutation sites in ApDAEase. **(A)** Superimposed view of the residues interacting with the metal ion from different DAEase enzymes that share sequence similarity. The yellow sticks with letter code A represent DAEase from *Arthrobacter psychrolactophilus* (ApDAEase), the cyan sticks with letter code B represent DAEase from *Arthrobacter globiformis*, and the green sticks with letter code C represent DAEase from *Agrobacterium tumefaciens*. **(B)** Surface electrostatic potential distribution of the protein. The color scale indicates the potential from − 6.0 kT/e (red, electronegative) to + 6.0 kT/e (blue, electropositive). The locations of residues H6, E35, S63, V105, and M110 within the pocket environment are highlighted. **(C)** Analysis of the hydrophobicity and interaction network surrounding the substrate-binding pocket. The protein backbone is shown in black, with red lines representing the interaction network. **(D)** Local flexibility analysis of the ApDAEase structure based on B-factor values. The color gradient represents the B-factor scale from 5Å^2^ (blue, rigid) to 50Å^2^ (red, flexible).

The structural prediction of ApDAEase utilizing AlphaFold indicates a tetrameric quaternary organization, which closely resembles the architecture found in experimentally determined structures of other ketose epimerase, including the DAEase from *Agrobacterium tumefaciens*^34^, and L-ribulose 3-epimerase from *Arthrobacter globiformis* (AgLREase)^15^. In each ApDAEase monomer, the residues E146, D179, H205, and E240 are anticipated to coordinate the probable Mg²⁺ ion at the active site, forming an arrangement similar to the metal coordination spheres documented for other epimerases (Fig. 5A). This configuration exhibits a significant degree of conservation within the DAEase family, as evidenced by the crystal structure of DAEase from *Agrobacterium tumefaciens* (AtDAEase), which demonstrated metal ion coordination through Glu150, Asp183, His209, and Glu244. In a comparable manner, the residues that coordinate the metal ion in AgLREase are identical to those found in ApDAEase, specifically Glu146, Asp179, His205, and Glu240. This observation underscores the conserved characteristics of metal coordination in these enzymes.

Previous reports reveal the mechanism of epimerization for DAEase functions via metal-dependent acid-base catalysis^15,35^. A divalent metal ion, coordinated by conserved residues such as glutamate, aspartate, and histidine, is crucial for substrate binding and the stabilization of the transition state. In general, the metal ion, commonly Mg²⁺ or Mn²⁺, interacts with the hydroxyl groups located at positions O1, O2, and O3 of d-fructose, securing the substrate in an ideal alignment within the active site^36–38^. Essential glutamate residues act as proton donors and acceptors, enabling proton abstraction and subsequent re-protonation at the stereocenter C3, with the catalytic process occurring via a cis-enediolate intermediate that is stabilized by the metal ion. The binding of the substrate induces conformational alterations in adjacent loop regions, effectively sealing the active site and facilitating accurate positioning for catalytic activity. Considering the essential function of the O1, O2, and O3 atoms along with their coordinating residues in the catalytic mechanism, it is prudent for engineering initiatives to refrain from altering these residues to maintain enzyme activity (Table S3). The conserved architecture of metal binding and the active site collectively support the catalytic mechanism that is characteristic of these enzymes. This study focused on residues that, while not directly interacting with the substrate, are located near the binding pocket. Residues positioned beyond the immediate substrate-interacting region (> 4.5 Å from the O4–O6 cluster) or exhibiting limited physicochemical variation relevant to substrate interaction were deprioritized. In contrast, H6, E35, S63, V105, and M110 were selected due to their location within the O4–O6 binding subsite, where they define the pocket boundary and provide diverse electrostatic, polar, and hydrophobic characteristics (Table S3).

The polarity of residues within the substrate binding pocket of DAEase significantly influences substrate specificity, binding affinity, and catalytic efficiency, as demonstrated in recent studies^15,35,39^. The characteristics of amino acid side chains play a crucial role in determining the formation of hydrogen bonds, electrostatic interactions, and the overall configuration and polarity of the binding site environment. Hydrophobic residues in proximity can facilitate the establishment of a more advantageous microenvironment for catalytic processes. Experimental mutagenesis and evolutionary studies demonstrate that the exchange of polar and nonpolar residues in or near the pocket influences both binding affinity and catalytic efficiency^40^. In this study, we aim to explore the influence of residue polarity within the binding pocket of DAEase on its catalytic activity. This will be achieved by altering the residues H6, E35, S63, V105, and M110 through the exchange of their polarity or electrostatic charge.

Furthermore, the electrostatic surface analysis conducted with the APBS plugin in PyMOL indicated that the residues H6, E35, S63, V105, and M110 are situated within a negatively polarized area in proximity to the O4–O6 substrate-binding cluster. The electrostatic environment is expected to affect substrate approach and orientation, given that electrostatic interactions are recognized for their role in directing long-range substrate steering and binding orientation within enzyme systems^12,41,42^. The analysis of hydrophobicity suggests that these residues constitute a chemically diverse interface, which includes both polar/charged residues (H6, E35, S63) and hydrophobic residues (V105, M110). The observed compositional diversity indicates that this region operates as a modifiable microenvironment at the periphery of the binding pocket. The structural arrangement of H6, E35, and S63 within β-sheet elements contributes to a relatively rigid framework. In contrast, V105 and M110 are situated in loop regions, which afford greater conformational flexibility. The integration of rigid and flexible structural components presents significant benefits, facilitating the modulation of pocket stability and local dynamics while maintaining the integrity of the catalytic core. The combined analysis of functional constraints and structural–physicochemical characteristics (Fig. 5B-D) supports the identification of H6, E35, S63, V105, and M110 as appropriate candidates for mutagenesis, with the objective of modulating substrate interaction while maintaining enzymatic stability.

The impact of residue polarity alteration in ApDAEase was clearly demonstrated through catalytic activity assays, revealing that the majority of variants (H6D, S63D, V105D, M110D, and M110H) displayed a significant decrease in activity relative to the wild-type enzyme, with H6D nearly completely losing its catalytic function. The nearly complete loss in activity observed in the H6D variant of ApDAEase is consistent with results from SaDAEase^40^, where it was demonstrated that H6 plays a crucial role in stabilizing the substrate O6 through van der Waals interactions. Notably, the mutation to tyrosine resulted in a complete loss of activity, whereas the substitution with phenylalanine preserved functionality^40^. The introduction of a negatively charged aspartate at this position in ApDAEase likely disrupts the stabilization and alters the electrostatic environment, which accounts for the reduction of activity observed. Similarly, the alteration of S63 to aspartate led to a decrease in efficiency (Fig. 3B), aligning with previous findings that indicated that modifications at this specific position (S63H, S63C) in SaDAEase hindered catalytic function. The observed parallels underscore the essential function of H6 and S63 in preserving the exact microenvironment of the binding pocket necessary for optimal catalytic activity in DAEases. Prior investigations into AgLRE (AgDAE was renamed AgLRE for its higher activity on L-ribulose^15)^, which exhibit significant similarity to ApDAEase, reveal that the residues V105 and M110 are integrated into a hydrophobic environment positioned at the entrance of the active site, referred to as the hydrophobic pocket. The observed reduction in activity in the V105D and M110H variants suggests that the transition from a hydrophobic to a polar environment at this position adversely impacts enzymatic function. This finding underscores the significance of the hydrophobic pocket in the recognition of fructose substrates, corroborating earlier results reported by Watanabe et al. (2024)^35^ and Zhu et al. (2019)^40^.

**Fig. 6:**
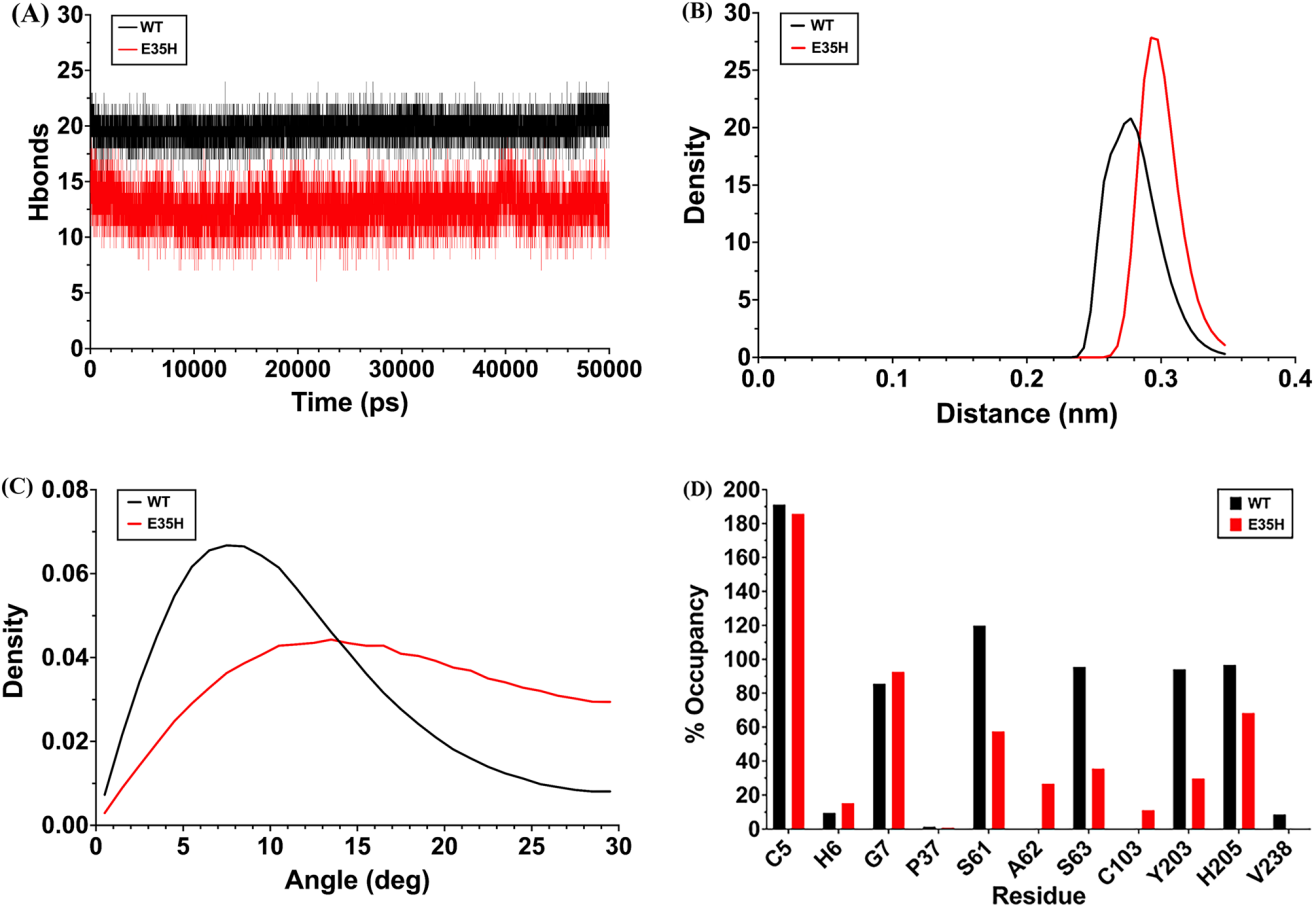
Comparative hydrogen bonding analysis of the residue 35 local environment in ApDAEase wild-type (WT) and E35H mutant. **(A)** Time-dependent evolution of the total number of hydrogen bonds formed at position 35 throughout the 50 ns MD simulation trajectories. **(B)** Probability density distribution of hydrogen bond distances (nm). **(C)** Probability density distribution of hydrogen bond angles (degrees). **(D)** Hydrogen bond occupancy (%) of neighbouring residues interacting with position 35. Wild-type (WT) and E35H mutant profiles are represented in black and red, respectively. Occupancy values exceeding 100% indicate residues involved in multiple concurrent hydrogen bond interactions.

**Fig. 7:**
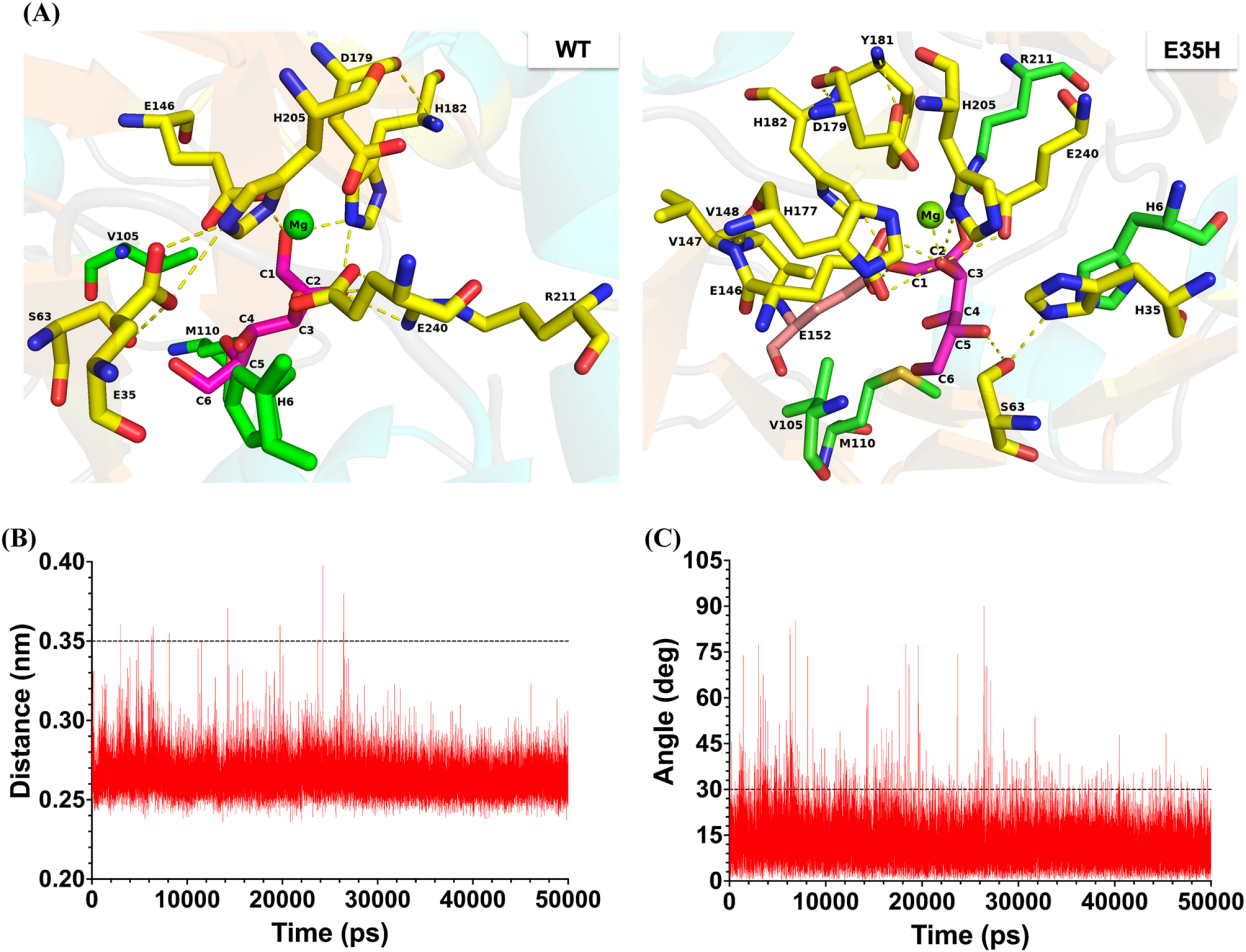
Structural and molecular dynamics analysis of substrate–residue interactions in ApDAEase wild-type and E35H mutant. **(A)** Key residue interactions of ApDAEase wild-type (left) and E35H mutant (right). Residues shown in yellow indicate those involved in interactions between key residues or with the substrate, while residues shown in green indicate those without such interactions. The Mg²⁺ ion is represented in green. **(B)** Time-dependent profile of the hydrogen bond distance (nm) between the residue E152 and the O1 atom of the substrate throughout the 50 ns MD simulation in ApDAEase E35H. **(C)** Time-dependent profile of the hydrogen bond angle (degree) for the same interaction in ApDAEase E35H. The horizontal dashed lines indicate the geometric criteria for hydrogen bond formation (distance ≤ 0.35 nm and angle ≤ 30°).

The improved catalytic performance of the E35H variant, demonstrating a 1.4-fold increase in catalytic efficiency relative to the wild-type, can be attributed to a carefully optimized interplay between local flexibility and overall structural rigidity. Kinetic analysis revealed a 1.3-fold reduction in *K*_m_, suggesting an increased affinity for the substrate, alongside a 1.1-fold rise in *k*_cat_, indicating a moderate enhancement in turnover rate. This enhancement is presumably linked to an increase in local flexibility within the microenvironment of the active site. In order to provide a deeper understanding of the mechanistic basis underlying this effect, additional molecular dynamics analyses were conducted to characterize the hydrogen-bond network with respect to persistence, geometry, and residue-level occupancy (Fig. 6A–D). The E35H variant demonstrates a diminished average number of hydrogen bonds (Fig. 6A) and decreased occupancy at multiple positions relative to the wild-type (Fig. 6D), suggesting a relaxation of local interaction constraints. The analyses of distance (Fig. 6B) and angle distributions (Fig. 6C) indicate a transition towards hydrogen-bond geometries that are marginally longer and exhibit greater variability, aligning with an enhancement in interaction flexibility. The hydrogen bonds in the E35H variant exhibit a transient nature rather than establishing rigid and persistent contacts, indicating an increase in local mobility within the binding pocket. The enhanced flexibility is expected to enable complex conformational changes necessary throughout the catalytic cycle, especially regarding substrate repositioning and product release, which in turn may account for the observed elevation in turnover number (*k*_cat_). An essential differentiation exists within the interaction networks. In the wild-type enzyme, the residues V105, M110, and H6 are not involved in hydrogen bonding with either the critical active-site residues or the substrate (Fig. 7A). In the E35H variant, these residues, in conjunction with R211, similarly exhibited no observable interactions. Nevertheless, a novel interaction was observed between E152 and the substrate O1, which presumably enhanced the anchoring and recognition of the substrate. To further validate this interaction, additional molecular dynamics analyses were conducted to monitor the distance and angular stability between E152 and the substrate O1 (Fig. 7B–C). The results demonstrate that the E152–O1 distance remains consistently within the hydrogen-bonding range (~ 0.25–0.30 nm) throughout the simulation, while the interaction angle is predominantly maintained below 30°, indicating a favorable and persistent geometry. These geometric criteria are widely used to define stable hydrogen bonds in molecular dynamics simulations^43^. Although minor fluctuations are observed, the interaction remains stable over time, suggesting that it is not a transient contact but a functionally relevant feature of the enzyme–substrate complex. This supplementary interaction offers a structural explanation for the heightened affinity noted in the reduced *K*_m_ values of E35H. Future studies involving site-directed mutagenesis of E152 (e.g., E152A) would be valuable to directly assess the functional contribution of this interaction. Disruption of this residue is expected to weaken substrate anchoring and may result in reduced catalytic performance, providing experimental validation of its role.

**Fig. 8:**
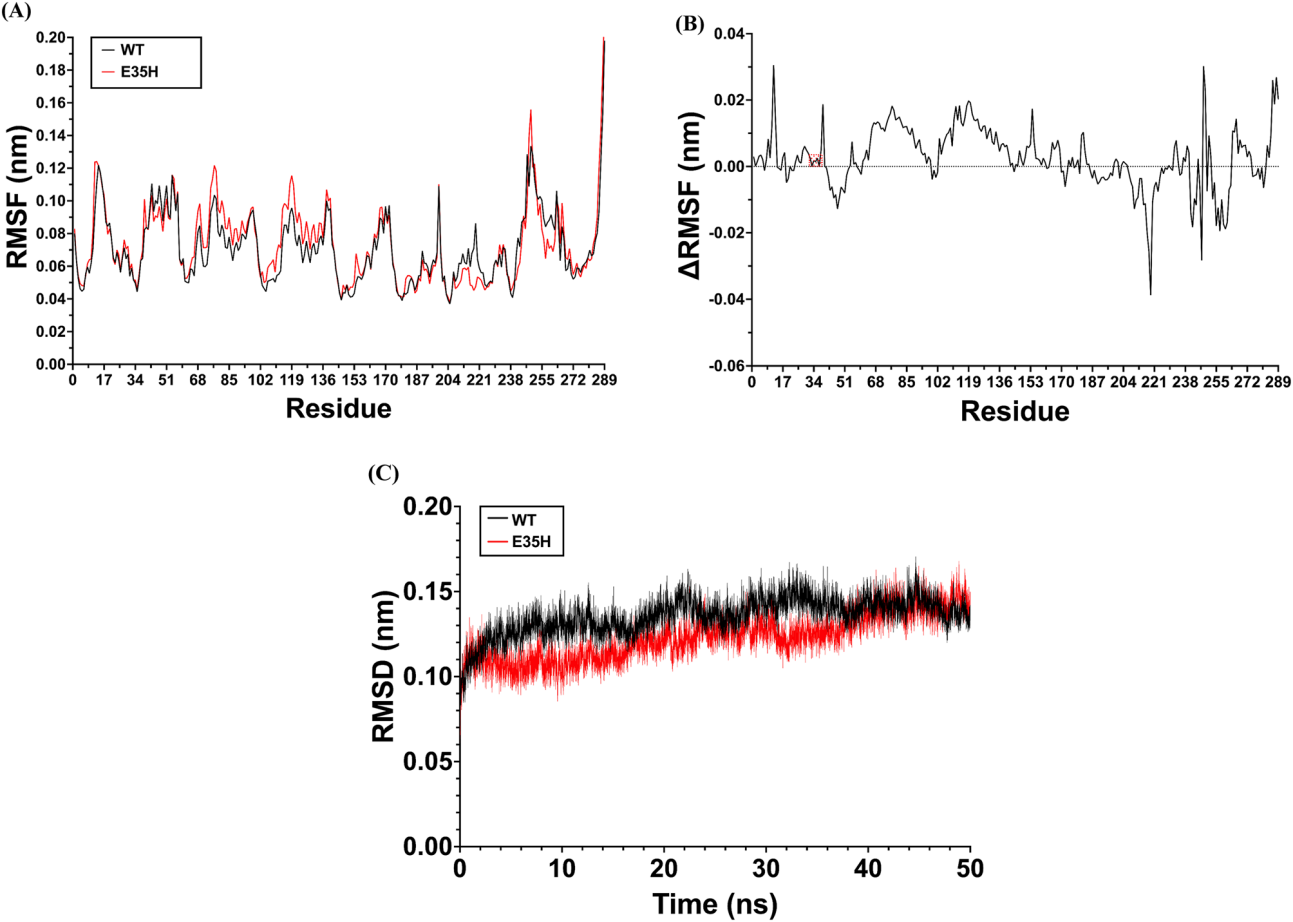
Comparative MD simulation analysis of ApDAEase wild-type and E35H mutant. (**A**) Root-Mean-Square fluctuation (RMSF) profiles of ApDAEase wild-type (black) and E35H mutant (red) across residues during molecular dynamics simulations. (**B**) Difference in RMSF (ΔRMSF) between wild-type and E35H mutant, highlighting regions with altered flexibility. (**C**) Root-Mean-Square Deviation (RMSD) profiles of the C-alpha atoms for the wild-type (black) and E35H mutant (red) as a function of simulation time.

The findings from molecular dynamics simulations provide additional evidence for this interpretation. Analyses of RMSF and ΔRMSF indicated specific increases in fluctuation at residues 35, implying a heightened flexibility in the active site (Fig. 8A-B). The elevated RMSF observed at residue 35 indicates a potential functional significance of local flexibility in the catalytic process. Situated adjacent to the entrance of the substrate-binding pocket, increased flexibility at this site may promote substrate access by allowing for transient enlargement of the pocket and enhance substrate alignment by permitting conformational modifications toward a catalytically favorable orientation. While residue 35 does not engage directly in the catalytic process, its function as a second-shell residue suggests that it influences the dynamic conditions surrounding the active site, thus indirectly facilitating the formation of the transition state. This interpretation aligns with established studies that emphasize the significance of conformational dynamics and localized flexibility in the regulation of enzyme catalysis^11,44^.

The experimental characterization demonstrated that the E35H variant displays enhanced structural stability. CD spectroscopy revealed a modest increase in helical content, rising from 3.0% in the wild-type to 3.5% in the E35H variant, suggesting that secondary structure organization is either preserved or improved. Furthermore, thermal unfolding analysis revealed a slight elevation in melting temperature (94.3 °C for E35H compared to 92.6 °C for the wild-type), thereby supporting the enhanced thermostability despite the diminished hydrogen-bond network at the site of mutation. The experimental observations are confirmed by molecular dynamics simulations, with the RMSD profiles (Fig. 8C) demonstrating that both systems maintain structural stability throughout the duration of the simulation. The E35H variant demonstrates slightly reduced RMSD values in comparison to the wild-type, indicating a more stable global conformational ensemble. This suggests that the mutation does not undermine the overall structural integrity, despite resulting in enhanced local flexibility within the active-site region. The simultaneous presence of increased global stability alongside localized flexibility creates optimal conditions for catalysis, facilitating dynamic modifications in the binding pocket while maintaining the structural integrity of the enzyme scaffold. To further validate these findings, the folding free energy changes (ΔΔG) predicted via the MAESTROweb server (Table S4) suggest a stabilizing effect for the E35H mutation, consistent with both circular dichroism and thermal stability data. Conversely, the remaining mutations were anticipated to exert mildly destabilizing effects and did not demonstrate enhanced catalytic activity.

Collectively, these results suggest that the E35H substitution improves catalytic efficiency via several interconnected mechanisms: it decreases hydrogen-bond rigidity at residue 35, introduces advantageous flexibility at residues 35, and establishes an extra stabilizing interaction between E152 and substrate O1. The interplay between localized flexibility and overarching constraints reflects earlier findings in various enzyme systems, where strategically altered polarity and the establishment of novel interactions improved catalytic performance while preserving structural integrity^45,46^.

Table 2 shows a comparison of DAEase after modification from various bacterial sources. The E35H mutation in ApDAEase exhibited a dual advantage: enhanced thermostability (Tm 94.3 °C compared to 92.6 °C in the wild-type) and a 1.34-fold increase in relative activity. This finding underscores the significance of nuanced alterations in polarity within the binding pocket, which can provide both catalytic and structural benefits. In comparison to previously documented variants in other DAEase homologs, the E35H substitution aligns with a wider pattern where targeted modifications at the active site or adjacent regions enhance enzyme flexibility and substrate recognition, while concurrently stabilizing the overall structure.

**Table 2:** Comparison of melting temperatures (Tm) and relative activities. Thermostability and activity profiles of ApDAEase (WT and E35H) compared to reported DAEases from various species.

Origin	Variant	Tm (^o^C)	Relative activity (%)	Variant	Tm (^o^C)	Relative activity (%)	Reference
*Arthrobacter psychrolactophilus*	WT	92.6	100	E35H	94.3	134.4	This study
*Rhodopirellula baltica*	WT	45.7	100	L144F	58.3	156.4	Mao et al., 2020^10^
*Halanaerobium congolense*	WT	73.0	100	R156C / K260C	74.5	345	Zhu et al., 2021^47^
*Clostridium bolteae*	WT	55.8	100	E6P	56.9	155.6	Wang et al., 2025^48^
*Arthrobacter globiformis* M30	WT	66.6	100	E75P / S137K / A200K / V237I / A270K / H13R / S245A / V34I / P172G / I176V / S218N / T223N	96.1	129	Shimada et al., 2025^49^
*Dorea* sp.	WT	56.6	100	F154Y / E191D / I193F	74.18	98.3	Zhang et al., 2018^50^

Mao et al. (2020)^10^ demonstrated that the L144F mutation in RbDAEase resulted in an increase in thermostability (Tm 58.3 °C compared to 45.7 °C in the wild-type) and enhanced catalytic performance (156.4% activity). In a similar vein, Wang et al. (2025)^48^ demonstrated that the E6P substitution in CbDAEase resulted in an increase in Tm of approximately 1.1 °C, alongside a significant enhancement in activity by 155.6%. Multi-site variants have demonstrated significant efficacy: Shimada et al. (2025)^49^ introduced several substitutions (e.g., E75P, S137K, A200K, etc.) in AgDAEase, resulting in a notable enhancement in stability (Tm 96.1 °C compared to 66.6 °C) and a 29% boost in activity. In a separate study, Zhu et al. (2021)^47^ observed that double mutants (R156C/K260C) in HcDAEase exhibited increased stability (Tm 74.5 °C compared to 73 °C) and significantly boosted activity (345%).

Within this framework, the E35H variant of *A. psychrolactophilus* is noteworthy as it attains a moderate yet well-rounded improvement in both stability and activity through a singular substitution, in contrast to extensive modifications involving multiple residues. In contrast to the significant activity enhancements observed in *H. congolense*^47^ or *C. bolteae*^48^, the 1.34-fold increase in catalytic efficiency of E35H indicates a more nuanced optimization of local hydrogen-bonding and flexibility. This aligns with previously documented advantageous single-site modifications, such as L144F in *R. baltica*^10^.

In summary, these comparisons highlight a widely applicable principle: modifications in polarity at critical binding-pocket sites, achieved through the introduction of hydrophobic characteristics (L144F)^10^, the disruption of hydrogen-bond networks (E6P)^48^, or the adjustment of charge balance (E35H), can lead to a combined enhancement in enzyme activity and thermal stability. The E35H variant contributes to an expanding collection of evidence indicating that strategically selected single-site mutations can yield considerable functional enhancements while circumventing the instability frequently linked to extensive mutagenesis.

It is crucial to emphasize that the structural interpretations provided in this study rely on models predicted by Alpha Fold 3 and subsequently refined through molecular dynamics simulations, as opposed to being derived from experimentally obtained crystal or cryo-EM structures. Alpha Fold 3 has exhibited exceptional capabilities in forecasting protein structures and elucidating molecular interactions and complexes^13^. However, the models produced should be viewed as predictive frameworks that inform mechanistic hypotheses. Our analysis of hydrogen-bond rearrangements, flexibility changes at residues 35 and 6, and the emergence of additional contacts in the E35H variant offers significant structural insight. However, further crystallographic or cryo-EM studies will be necessary to experimentally validate these predictions.

## Conclusion

The present study reveals that the strategic engineering of ApDAEase by adjusting charge and polarity in the active-site microenvironment can substantially enhance catalytic efficiency while maintaining thermostability. Among the engineered variants, the E35H substitution distinctly improved enzymatic properties, resulting in a 1.34-fold increase in activity, a 1.3-fold decrease in *K*_m_, and a 1.1-fold increase in *k*_cat_, culminating in a 1.4-fold enhancement in catalytic efficiency relative to the wild-type enzyme. Through structural and biophysical analyses, it was determined that the E35H variant exhibits an optimal balance of localized flexibility and global rigidity, enhanced by the establishment of a stabilizing interaction between E152 and the substrate O1. This mutation notably resulted in a slight increase in thermostability (Tm 94.3 °C compared to 92.6 °C in the wild-type), highlighting the combined benefits of improved catalytic activity and enhanced thermal stability. This study establishes a framework for the targeted engineering of epimerases, thus aiding in the advancement of reliable and effective biocatalysts for the large-scale industrial production of d-allulose.

## Supplementary Information

Below is the link to the electronic supplementary material.

Supplementary Material

## Data Availability

The datasets analysed during the current study for ApDAEase are available in the NCBI repository for amino acid sequence (Accession No. WP_110484351.1, https://www.ncbi.nlm.nih.gov/protein/WP_110484351.1) and nucleotide sequence (Accession No. NZ_QJVC01000003.1, https://www.ncbi.nlm.nih.gov/nuccore/NZ_QJVC01000003.1). The protein record is also available in the UniProtKB database (Entry code A0A2V5JHE2, https://www.uniprot.org/uniprotkb/A0A2V5JHE2/entry).
