# Supplementary material for: One-site polarity switch enhances catalytic efficiency and stability of d-allulose 3-epimerase via flexibility–rigidity rebalancing
Source: Sci Rep. 2026 May 30;16:24811. doi: 10.1038/s41598-026-54479-z (PMC13458763; doi:10.1038/s41598-026-54479-z)
Supplement: Supplementary file 1 [file 41598_2026_54479_MOESM1_ESM.docx]

**Table S1. Primers used for site-directed mutagenesis of ApDAEase**

| Primer | Oligonucleotide sequence | | Usage |
| --- | --- | --- | --- |
|  | Forward (5’ to 3’) | Reverse (5’ to 3’) |  |
| H6D | G AAA ATT GGT TGC GAC GGT CTG GTA TGG ACC GGT | ACC GGT CCA TAC CAG ACC GTC GCA ACC AAT TTT C | Mutagenesis at residue 1038-1071 |
| E35H | GCA GGC TTT GAC CTG ATC CAT TTT CCT TTG ATG GAC CC | GG GTC CAT CAA AGG AAA ATG GAT CAG GTC AAA GCC TGC | Mutagenesis at residue 952-989 |
| S63D | GTT TCT GCG GAT TTG GGT CTC TCG | CGA GAG ACC CAA TAG CGC AGA AAC | Mutagenesis at residue 873-896 |
| V105A | GGC ACC GAA TTT TGT GGT GCC ATT TAC AGC TC | GA GCT GTA AAT GGC ACC ACA AAA TTC GGT GCC | Mutagenesis at residue 748-779 |
| V105D | C GGC ACC GAA TTT TGT GGT TAG ATT TAC AGC TCC ATG AAG AAG TAC ATG G | C CAT GTA CTT CTT CAT GGA GCT GTA AAT ATC ACC ACA AAA TTC GGT GCC G | Mutagenesis at residue 731-780 |
| M110D | CGA ATT TTG TGG TGT CAT TTA CAG CTC CGA CAA GAA GTA CAT GGA GCC AC | GTG GCT CCA TGT ACT TCT TGT CGG AGC TGT AAA TGA CAC CAC AAA ATT CG | Mutagenesis at residue 731-774 |
| M110H | CGA ATT TTG TGG TGT CAT TTA CAG CTC CCA TAA GAA GTA CAT GGA GCC AC | GTG GCT CCA TGT ACT TCT TAT GGG AGC TGT AAA TGA CAC CAC AAA ATT CG | Mutagenesis at residue 731-774 |

**Table S2. Thermal melting data of ApDAEase wild-type and E35H mutant obtained from Spectra Manager^TM^ software (JASCO J-1500)**

| No | Sample | Tm [deg C] | Tm (error: +/-) | dH [J/mol] | dH (error: +/-) | dS [J/mol/K] | dS (error: +/-) |
| --- | --- | --- | --- | --- | --- | --- | --- |
| 1 | Wild-type | 92.62 | 0.878642 | 170653 | 14507.8 | 466.564 | 39.6641 |
| 2 | E35H | 94.33 | 1.15043 | 143703 | 15267 | 391.055 | 41.5456 |

**Table S3. Rational selection and exclusion criteria for ApDAEase mutagenesis**

| Selection Filter | Residue Category | Location | Status | Rationale for Selection/Exclusion |
| --- | --- | --- | --- | --- |
| Metal Coordination | E146, D179, H205, E240 | Active site | Excluded | Essential for Mg^2+^ cofactor binding |
| Substrate Anchoring | H182, R211 | Active site | Excluded | Involved in direct hydrogen bonding with the O1, O2, and O3 atoms of the substrate (Fig 1A) |
| Design Target (O4-O6) | H6, E35, S63, V105, M110 | Binding pocket (<4.5Å from substrate) | Selected | Selected based on representative sampling of physicochemical diversity (aromatic, electrostatic, hydrogen-bonding, and hydrophobic interactions) within the O4–O6 binding subsite to explore varied substrate-enzyme complementarity |

**Table S4. Predicted folding free energy changes (ΔΔG) for ApDAEase variants using MAESTROweb**

The stability impacts of single-point mutations were predicted using the MAESTROweb server (<https://pbwww.services.came.sbg.ac.at/maestro/web>). A negative ΔΔG value indicates a stabilizing effect, while a positive value indicates a destabilizing effect on the protein structure.

| Mutant | Substitution | Predicted ΔΔG (kcal/mol) | Stability Impact |
| --- | --- | --- | --- |
| H6D | His to Asp | 0.240 | Destabilizing |
| E35H | Glu to His | - 0.205 | Stabilizing |
| S63D | Ser to Asp | 0.123 | Destabilizing |
| V105D | Val to Asp | 0.444 | Destabilizing |
| M110D | Met to Asp | 0.509 | Destabilizing |
| M110H | Met to His | 1.116 | Destabilizing |

**Figure S1. Multiple sequence alignment of DAEases from various microbial sources**

The alignment highlights the high degree of conservation between D-allulose 3-epimerase from *Arthrobacter psychrolactophilus* (ApDAEase) and its homologs. Strictly conserved residues are boxed in red. Key functional residues within the active site are indicated by symbols below the alignmen. Circles (●): Represent residues involved in metal coordination. Triangles (**▲**): Represent substrate anchoring residues responsible for direct hydrogen bonding with the substrate.

**Figure S2. Structural validation and confidence metrics of AlphaFold-predicted ApDAEase**

(A) Wild-type (WT) ApDAEase and (B) E35H mutant. The 3D structures are colored according to the Predicted Local Distance Difference Test (pLDDT) scores, with dark blue indicating very high confidence (pLDDT > 90). The high ipTM and pTM scores of 0.96 (shown for WT) reflect excellent accuracy in both the global fold and the relative orientation of the subunits.

**(A)**

**(B)**

**Figure S3. Original Uncropped Gel for Figure 1B**

Full-length gel electrophoresis of PCR products for ApDAEase mutants. The uncropped gel shows the successful amplification of mutant candidates. Lane M: DNA ladder (GeneRuler 1 kb DNA ladder); Lane 1: S63D mutant; Lane 2: V105D mutant; Lane 3: H6D mutant; Lane 4: M110H mutant; Lane 5: M110D mutant; Lane 6: E35H mutant; Lane X: unused lane.

**Figure S4. Original Uncropped Gel for Figure 2A_1**

Full-length SDS-PAGE gels of ApDAEase mutants purification. The uncropped gels show the protein profiles during the purification process. Lane M: Protein ladder; Lane C: Crude extract; Lane P: Purified protein. Lane X: Unused lane.

**Figure S5. Original Uncropped Gel for Figure 2A_2**

Full-length SDS-PAGE gels of ApDAEase mutants purification. The uncropped gels show the protein profiles during the purification process. Lane M: Protein ladder; Lane C: Crude extract; Lane P: Purified protein. Lane X: Unused lane.

**Figure S6. Original Uncropped Gel for Figure 2A_3**

Full-length SDS-PAGE gels of ApDAEase mutants purification. The uncropped gels show the protein profiles during the purification process. Lane M: Protein ladder; Lane C: Crude extract; Lane P: Purified protein. Lane X: Unused lane.

**Figure S7. Original Uncropped Gel for Figure 2B**

Full-length Western blot of ApDAEase mutants. The uncropped blot image shows the specific detection of ApDAEase mutants. Lane M: Protein ladder; Lane X: Unused lane.
